# Molecular epidemiology and hematologic characterization of δβ-thalassemia and hereditary persistence of fetal hemoglobin in 125,661 families of greater Guangzhou area, the metropolis of southern China

**DOI:** 10.1186/s12881-020-0981-x

**Published:** 2020-02-28

**Authors:** Fan Jiang, Liandong Zuo, Dongzhi Li, Jian Li, Xuewei Tang, Guilan Chen, Jianying Zhou, Hang Lu, Can Liao

**Affiliations:** 1 The Second School of Clinical Medicine, Southern Medical University, Guangzhou, Guangdong Province People’s Republic of China; 2 Prenatal Diagnostic Center, Guangzhou Women and Children’s Medical Center affiliated with Guangzhou Medical University, Guangzhou, Guangdong Province People’s Republic of China

**Keywords:** Prevalence, δβ-thalassemia, HPFH, Guangzhou

## Abstract

**Background:**

Individuals with δβ-thalassemia/HPFH and β-thalassemia usually present with intermedia or thalassemia major. No large-scale survey on HPFH/δβ-thalassemia in southern China has been reported to date. The purpose of this study was to examine the molecular epidemiology and hematologic characteristics of these disorders in Guangzhou, the largest city in Southern China, to offer advice for thalassemia screening programs and genetic counseling.

**Methods:**

A total of 125,661 couples participated in pregestational thalassemia screening. 654 subjects with fetal hemoglobin (HbF) level ≥ 5% were selected for further investigation. Gap-PCR combined with Multiplex ligation dependent probe amplification (MLPA) was used to screen for β-globin gene cluster deletions. Gene sequencing for the promoter region of HBG1 /HBG2 gene was performed for all those subjects.

**Results:**

A total of 654 individuals had hemoglobin (HbF) levels≥5, and 0.12% of the couples were found to be heterozygous for HPFH/δβ-thalassemia, including Chinese ^G^γ (^A^γδβ)^0^-thal, Southeast Asia HPFH (SEA-HPFH), Taiwanese deletion and Hb Lepore–Boston–Washington. The highest prevalence was observed in the Huadu district and the lowest in the Nansha district. Three cases were identified as carrying β-globin gene cluster deletions, which had not been previously reported. Two at-risk couples (0.0015%) were required to receive prenatal diagnosis. We also found 55cases of nondeletional-HPFH (nd-HPFH), including 54 with Italian nd-HPFH and one with the ^A^γ-197C-T heterozygous state. It is difficult to discriminate between Chinese ^G^γ (^A^γδβ)^0^-thal and Italian nd-HPFH carriers using hemoglobin (Hb) analysis.

**Conclusions:**

This study is the first to describe the familial prevalence of HPFH/δβ-thalassemia and the high-risk rate in Greater Guangzhou Area, and the findings will support the implementation of thalassemia screening for three common deletions by gap-PCR. We also presented a systematic description of genotype-phenotype relationships which will be useful for genetic counseling and prenatal diagnostic services for β-thalassemia intermedia.

## Background

Thalassemias (thals) comprise a group of monogenic diseases that are highly common in such locations as the Mediterranean region, North Africa, and Southern China. Thalassemias include α-, β-, delta-beta-thalassemia (δβ-thalassemia) and hereditary persistence of fetal hemoglobin (HPFH),as determined by the type of globin chain that is involved [1]. The characteristic of δβ-thalassemia and HPFH is high fetal hemoglobin (HbF) levels in adults. δβ-Thalassemia (OMIM #141749, ORPHA:231237) results from deletions in the δ-globin and β-globin gene regions, but the γ-globin genes are rarely involved [2]. HPFH (OMIM #141749, ORPHA:46532) includes deletional HPFH and nondeletional HPFH (nd-HPFH), and the former is caused by large deletions in the region between the γ- and β-globin genes [3]. It is not always possible to distinguish between HPFH and delta-beta-thalassemia because the distinction between them is subtle. Deletional HPFH/δβ-thalassemia typically results in a clinical phenotype with microcytic hypochromic anemia. Moreover, coinheritance with minor β-thalassemia or Hb variants in the β-globin chain has been identified to cause thalassemia intermedia or major, although clinical phenotypes vary [4–8]. In general, the identification of carriers plays an important role in prenatal diagnosis, especially in regions with high β-thalassemia occurrence. More than 50 HPFH/δβ-thalassemia deletions have been reported to date, which are related to different ethnic backgrounds (http://globin.cse.psu.edu/hbvar/menu.html). The Chinese^G^γ (^A^γδβ)^0^ (NG_000007.3:g.48795_127698del78904) and SEA-HPFH (NC_000011.9: g.5222878_5250288del) maybe the most common β-globin cluster deletions in China. Table 1 lists those two β-globin cluster deletions and their associated phenotypes in southern China. The prevalence of deletional HPFH/δβ-thalassemia was reported in Yunnan, Guangdong and Guangxi provinces [9, 10]. This prevalence was highest in the Guangxi Zhuang Autonomous Region, reaching 0.21%, while it was lower than 0.002% in Hakka People living in the Guangdong Meizhou areas [11]. Cases of other types of deletional HPFH/δβ-thalassemia, such as Taiwanese deletion (NC_000011.9:g.5247493_5248849del) and Hb Lepore, have been reported as cases in China. Five variants of Hb Lepore have been reported due to different gene deletion breakpoints. All these variants were found in different areas, such as Hb Lepore-Hollandia (HGVS: NG_000007.3: g.63290_70702del), which has been reported in people of southern and southeast Asia, with rare occurrences being observed in Europe [12].

**Table 1:** The most commonly known HPFG/δβ-thalassemia and their associated phenotypes in Southern China

Name	Type of Thalassemia	Mutation	Phenotype
Chinese ^G^gamma (^A^gammadeltabeta)^0^-Thal	(Agamma-delta-beta)^0^	NG_000007.3:g.48795_127698del78904	Microcytic anemia (HP:0001903); Persistence of hemoglobin F (HP:0011904);
SEA-HPFH deletion	HPFH	NC_000011.9:g.5222878_5250288del27411	Microcytic anemia (HP:0001903);Persistence of hemoglobin F (HP:0011904);

Point mutations or small deletions in the proximal fetal γ-globin gene promoter lead to nd-HPFH. However, as individuals with nd-HPFH are usually healthy, HPFH appears to be a natural and benign mechanism to elevate HbF expression in adults [13]. Although considerable research has recently been focused on nd-HPFH in efforts to reactivate the fetal γ-globin gene in adulthood for β-thalassemia treatment [14–16], there is little information regarding the prevalence and hematologic characteristics of nd-HPFH in China, which would be helpful for precise genetic counseling.

Guangzhou is the capital of Guangdong Province and the largest city in southern China, with a native population of approximately 14.9 million. Although a free thalassemia screening program has been offered to pregestational couples in this region, carrier screening for deletional HPFH/δβ-thalassemia is not included in this program. Accordingly, no large-scale survey on the molecular epidemiology and hematological characteristics of deletional HPFH/δβ-thalassemia in southern China has been reported. To provide data to support thalassemia screening and enhance prevention efficiency, the aim of this study was to determine the molecular epidemiological characteristics and hematological data of deletional HPFH/δβ-thalassemia in Guangzhou. In addition, this study is the first of its kind to investigate the prevalence and molecular characterization of common nd-HPFH in this region, which is important for accurate thalassemia genetic counseling and prenatal diagnosis and for identifying new strategies for thalassemia therapy.

## Methods

### Human subjects

This study was approved by the Ethics Committee of the Guangzhou Women & Children Medical Center,and informed consent was obtained from all subjects participating in the study. In total, 125,661 couples participated in the pregestational thalassemia screening program from January 2016 to December 2018. Two milliliters of EDTA-anticoagulant-treated peripheral blood was collected for analysis of hemoglobin components and levels using capillary electrophoresis (CE; Serbia, Paris, France). Samples were collected for further screening of α- and β-thalassemia when at least one partner had a low MCV (<82 fL) or MCH lower than 27 pg [17].

### Genetic analysis

We extracted genomic DNA from the peripheral blood samples using a genomic DNA isolation kit (Qiagen; Hilden, Germany). Gap-PCR was used to detect common α-thalassemia deletions, including --SEA, −α3.7, −α4.2 and --Thailand. Three nondeletional α-thalassemia mutations and 17 known β-globin gene mutations were assessed using reverse dot-blot hybridization (RDB). All these assays were performed with a thalassemia gene detection kit (Shenzhen Yishengtang Biological Products Co., Ltd.; Shenzhen, China). Gap-PCR was performed for individuals with HbF levels ≥5% to screen two types of β-globin gene cluster deletions, Chinese (^A^γδβ)^0^ thalassemia and Southeast Asian (SEA) HPFH, using specific primers [18]. When the two common deletions were excluded, multiplex ligation-dependent probe amplification (MLPA) was employed to detect copy number variation in the β-globin gene cluster according to the manufacturer’s instructions (MRC Holland, Amsterdam, the Netherlands). Gap-PCR and sequence analysis using respective flanking primers were performed to determine deletional breakpoints. Sanger sequencing was applied to examine the promoter regions of γ-globin genes (including ^A^γ- and ^G^γ-globin genes) for all subjects with HbF levels ≥5%.

### Statistical analysis

The hematological parameters of subjects with different genotypes were described using the mean and standard deviation. Differences in hematological parameters between groups were compared by the nonparametric Mann-Whitney U test. The carrier rate of common deletional HPFH/δβ-thalassemias in 11 districts was analyzed by Pearson Chi-square analysis. A *p*-value of less than 0.05 was considered to indicate a significant difference.

### Method of drawing

The district map of Guangzhou was generated using ArcGIS Online (https://www.arcgis.com/index.html).

## Results

**Fig. 1:**
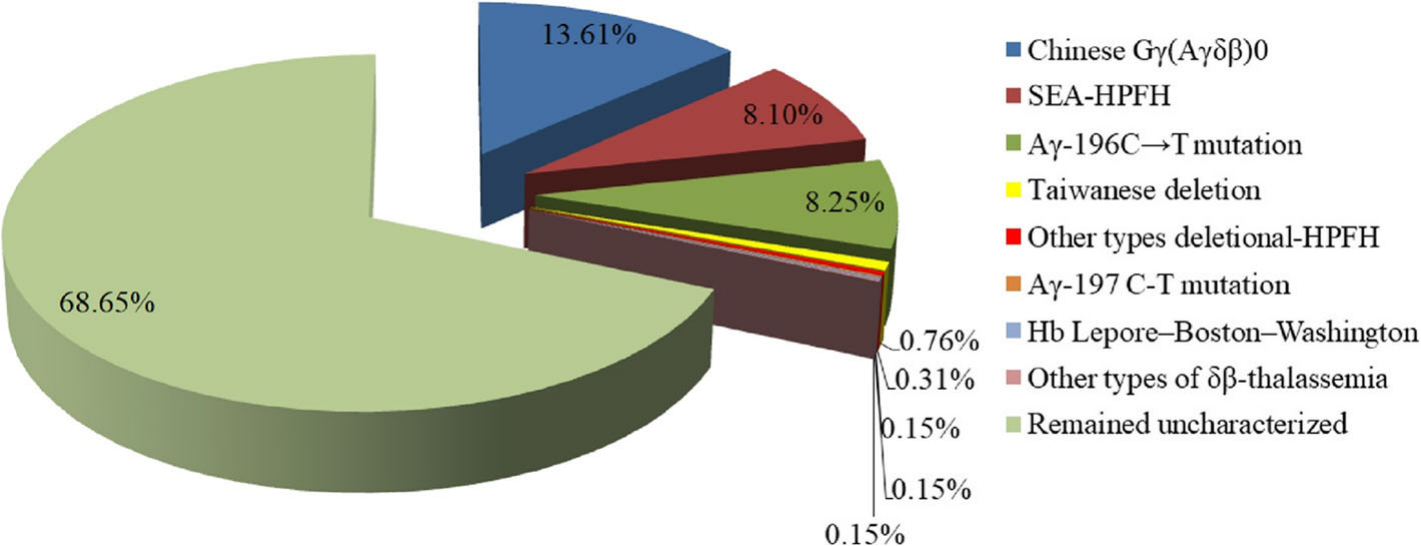
Spectrum of δβ-thalassemia and Hereditary Persistence of Fetal Hemoglobin in 654 subjects with HbF>5%

**Fig. 2:**
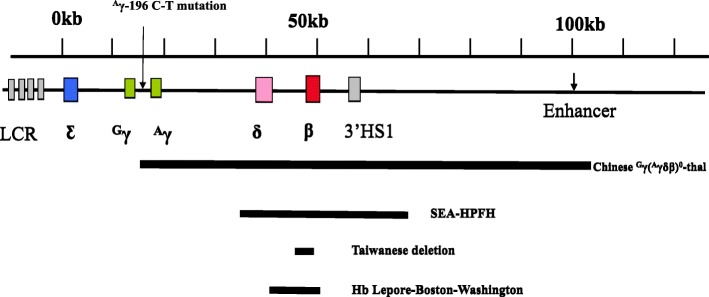
Structures of the common deletions and the locus of the (^A^γ-196 C-T) mutation identified, LCR, locus control region

**Fig. 3:**
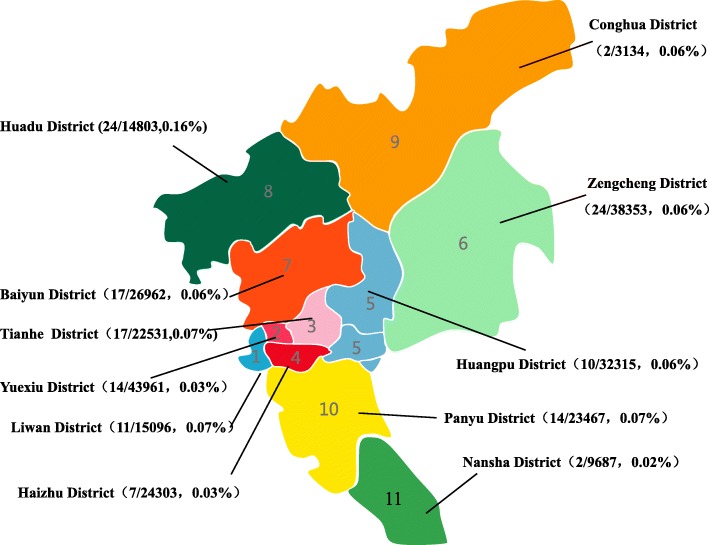
Map showing the prevalence of Chinese ^G^γ (^A^γδβ)^0^ thalassemia and SEA-HPFH in Eleven districts of Guangzhou. (The number and percentage of those two types of δβ thalassemia/HPFH are indicated in detail). The carrier rate of these two disorders in eleven districts of Guangzhou is shown in Fig. 3. It was 0.06% in Guangzhou, which was similar in most districts except for Huadu and Nansha.

A total of 654 individuals, including 277 men and 377 women aged 21 to 49 years from eleven districts of Guangzhou, were found to have a high percentage of HbF(> 5%). The most common deletional HPFH/δβ-thalassemia was Chinese ^G^γ (^A^γδβ)^0^ thalassemia, followed by SEA-HPFH. One hundred forty-two (142/654 21.71%) individuals had one of those two types mentioned above. We detected 5 cases of Taiwanese deletion (5/654, 0.76%) and one case of Hb Lepore. Three cases of β-globin gene cluster deletions (3/654, 0.46) not reported to date were identified by MLPA. We also identified 55 cases as non-deletional HPFH, including 54 samples with the ^A^γ-196 C-T mutation (53/654,7.95%), named Italian nd-HPFH, and one sample with ^A^γ-197 C-T. The spectra of HPFH/δβ-thalassemia are shown in Fig. 1.The hematological data of individuals with the main genotypes shown in Fig. 1 are presented in Table 2. Structures of the four common deletions and the locus of the^A^γ-196 C-T mutation are described in Fig. 2. The main aim of the thalassemia prevention and control program is based on the family as a unit. All couples with high HbF levels came from different families, therefore, we can determine the familial prevalence (the carry rate that one partner has δβ-thalassemia or Hereditary Persistence of Fetal Hemoglobin) was observed among 125,661 families. The familial prevalence of Chinese ^G^γ (^A^γδβ)^0^ thalassemia and SEA-HPFH were 0.071% (89/125661) and 0.042% (53/125661), respectively. Two of the 125,661 couples were identified as being at risk of having a baby with thalassemia intermedia. The carrier rate of these two disorders in eleven districts of Guangzhou is shown in Fig. 3**.** It was 0.06% in Guangzhou, which was similar in most districts except for Huadu and Nansha. The carrier rate was 0.16% in Huadu District, while it was only 0.03% in Nansha District. The Nansha district was established in 2005, with immigrants predominating. Approximately 76.89% of families immigrated to Nansha district from other places in our study. As the outer suburb, the migrant population is less than 30% in Huadu district.

**Table 2:** The hematological datas of individuals with the main genotype shown in Fig. 1

cases	Hb (g/dL)	MCV (fL)	MCH (pg)	HbA (%)	HbA2 (%)	HbF(%)	Genotype
77(F:49, M:28)	135.2 ± 14.9	70.6 ± 7.0	24.9 ± 15.3	82.0 ± 2.6	2.4 ± 0.3	15.5 ± 2.6	Chinese ^G^γ (^A^γδβ)^0^/β^N^
8 (F:1, M:7)	129.9 ± 8.37	69.1 ± 3.4	22.5 ± 1.4	86.8 ± 2.5	2.5 ± 0.2	10.6 ± 2.4	Chinese ^G^γ (^A^γδβ)^0^combined with --^SEA^/αα
1(M)	155	77.9	24.1	86.1	2.7	1.2	Chinese ^G^γ (^A^γδβ)^0^combined with -α^3.7^/αα
1(F)	126	81	26	82.1	2	15.9	Chinese ^G^γ (^A^γδβ)^0^combined with -α^4.2^/αα
1(M)	137	74.7	23.1	83.6	2.7	13.7	Chinese ^G^γ (^A^γδβ)^0^ combined withαα^WS^/αα
1(M)	128	64.8	20.3	16.0	2.7	81.3	Chinese ^G^γ (^A^γδβ)^0^/β^−28^
50(F:23,M:27)	137.8 ± 14.0	76.6 ± 4.3	25.1 ± 1.5	74.2 ± 4.2	4.0 ± 0.6	21.8 ± 4.8	SEA-HPFH/β^N^
3(F:1,M:2)	130.0 ± 11.1	69.5 ± 0.6	22.2 ± 0.2	76.4 ± 4.1	3.7 ± 0.5	19.9 ± 4.1	SEA-HPFH/β^N^ combined with --^SEA^/αα
5(F:2,M:3)	115.33 ± 23.75	66.73 ± 6.02	20.53 ± 2.76	86.26 ± 2.63	6.83 ± 0.57	6.9 ± 3.1	Taiwanese deletion/β^N^
37(F:15,M:22)	147.8 ± 14.6	88.7 ± 5.0	29.6 ± 2.1	85.0 ± 1.4	1.8 ± 0.2	14.9 ± 2.3	^A^γ-196 C-T mutation
14(F:7,M:7)	128.3 ± 18.9	68.3 ± 4.2	21.7 ± 1.2	83.4 ± 2.4	1.4 ± 0.1	13.8 ± 1.5	^A^γ-196 C-T mutation combined with --^SEA^/αα
1(F)	117	69.2	22.1	70	4.1	25.9	^A^γ-196C-T mutation combined with --^SEA^/αα and β^−28^/β^N^
1(F)	114	67.7	21.4	70	1.6	16.6	Homozygote for ^A^γ-196C-T mutation combined with --^SEA^/αα
1(M)	163	84	29.4	82.2	2.3	15.5	Homozygote for ^A^γ-196C-T mutation
1(M)	148	72.4	23.1	88.8	2.1	9.1	^A^γ-197 C-T mutationcombined with --^SEA^/αα
1(F)	108	72.8	23.8	77.2	2.3	11.30	Hb Lepore-Boston/Washington

**Fig. 4:**
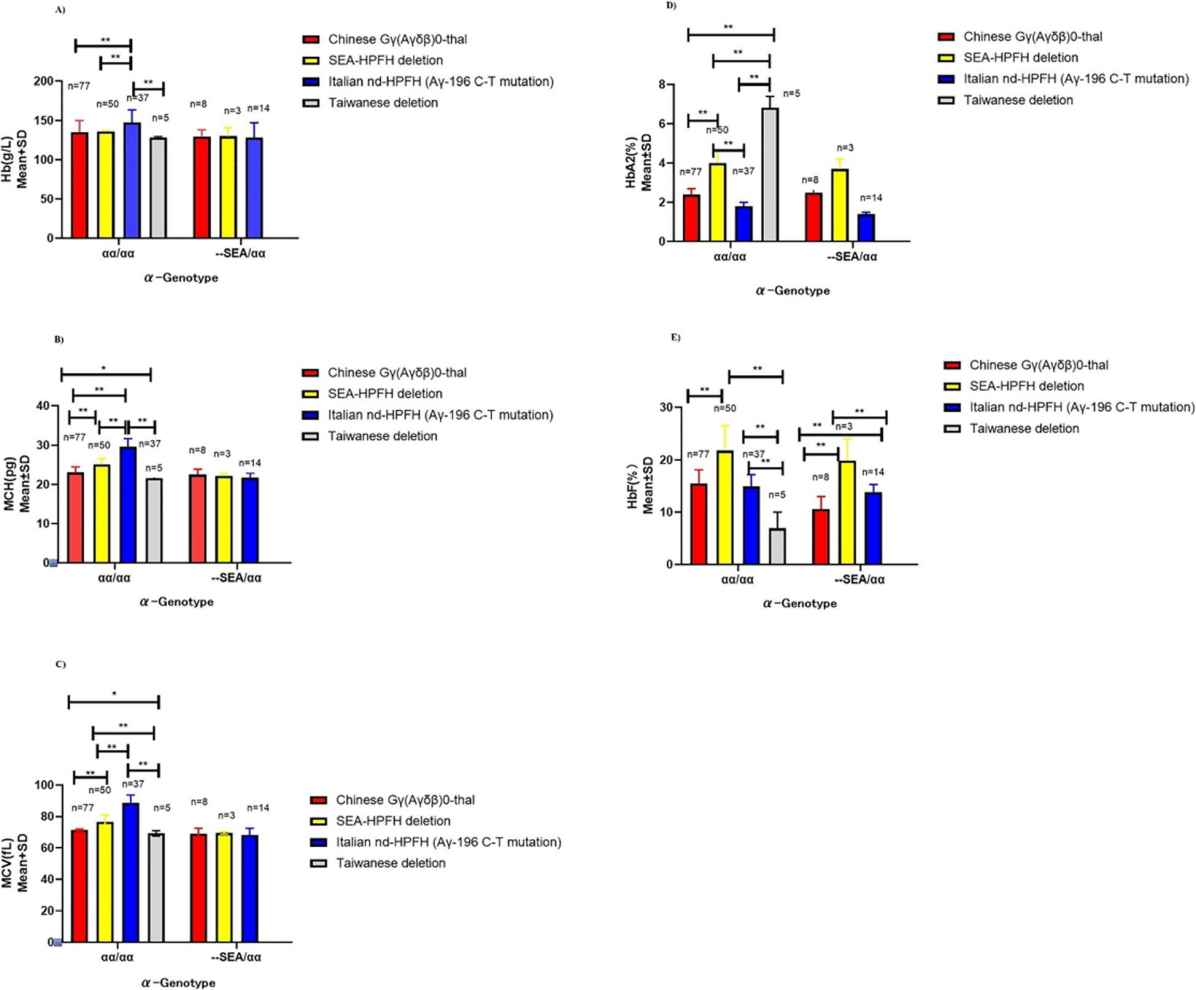
Hematological parameter analysis of heterozygotes of Chinese ^G^γ (^A^γδβ)^0^ thalassemia, SEA-HPFH, Taiwanese deletion, and Italian nd-HPFH. Comparisons of hemoglobin (Hb) (**a**), mean corpuscular volume (MCV) (**b**), mean corpuscular hemoglobin (**c**), hemoglobin A2 (HbA2) (**d**), and fetal hemoglobin (HbF) (**e**) levels of those four types of δβ-thalassemia/HPFH with or without coinherited α^0^-thalassaemia (southeast Asian type deletion). Mean ± standard deviation (SD) isused to describe hematological parameters. A *p*-value of less than 0.05 showing significant differences: **p < 0.01; *p < 0.05

**Fig. 5:**
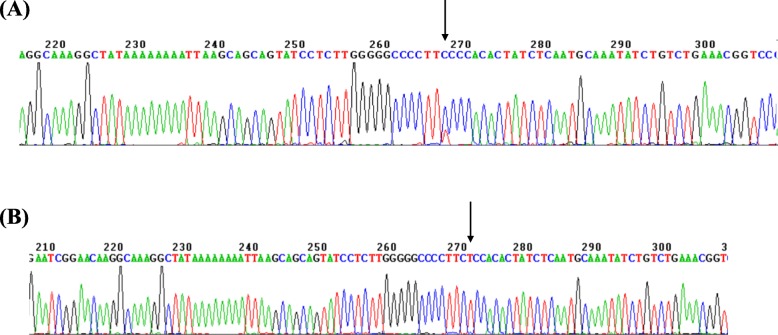
Chromatograms of point mutations in the proximal fetal γ-globin gene promoters detected in individuals. (**a**) Cretan HPFH (^A^γ-197 C-T); (**b**) Homozygote for (^A^γ-196 C-T) mutation

Among 89 Chinese ^G^γ (^A^γδβ)^0^ thalassemia mutations, 77 were in the heterozygous state; 11 were combined with α-thal and one was combined with β-thal. Three cases of coinheritance of SEA-HPFH and SoutheastAsia deletional type (−- ^SEA^ /αα) were detected. We also observed 14 individuals with coinherited Southeast Asia deletional type (−-^SEA^/αα) and ^A^γ-196C-T mutation. The hematological data are shown in Fig. 4. For the first time, we describe two homozygous carriers of the ^A^γ-196 C-T mutation and one with coexistence of α^0^β-thal and the ^A^γ-196C-T mutation. (Fig. 5a). Another nondeletional HPFH variant named Cretan HPFH (^A^γ-197 C-T mutation) was detected for the first time in China as well. This individual was also a carrier of the Southeast Asia deletional type (−-^SEA^/αα) (Fig. 5b). The hematological analysis showed high HbF levels and hypochromic microcytic parameters (MCV72.4 fL, MCH 23.1 pg, Hb 148 g/L, Hb A 88.8%, Hb A2 2.1%, HbF 9.1%).

The individual with Hb Lepore showed a Hb variant in Zone 6, for a level of 8.6% by CE **(**Supplementary File 1 A). Gap-PCR and MLPA were employed to confirm Hb Lepore–Boston–Washington (Supplementary Files 1 B and C).

**Fig.6:**
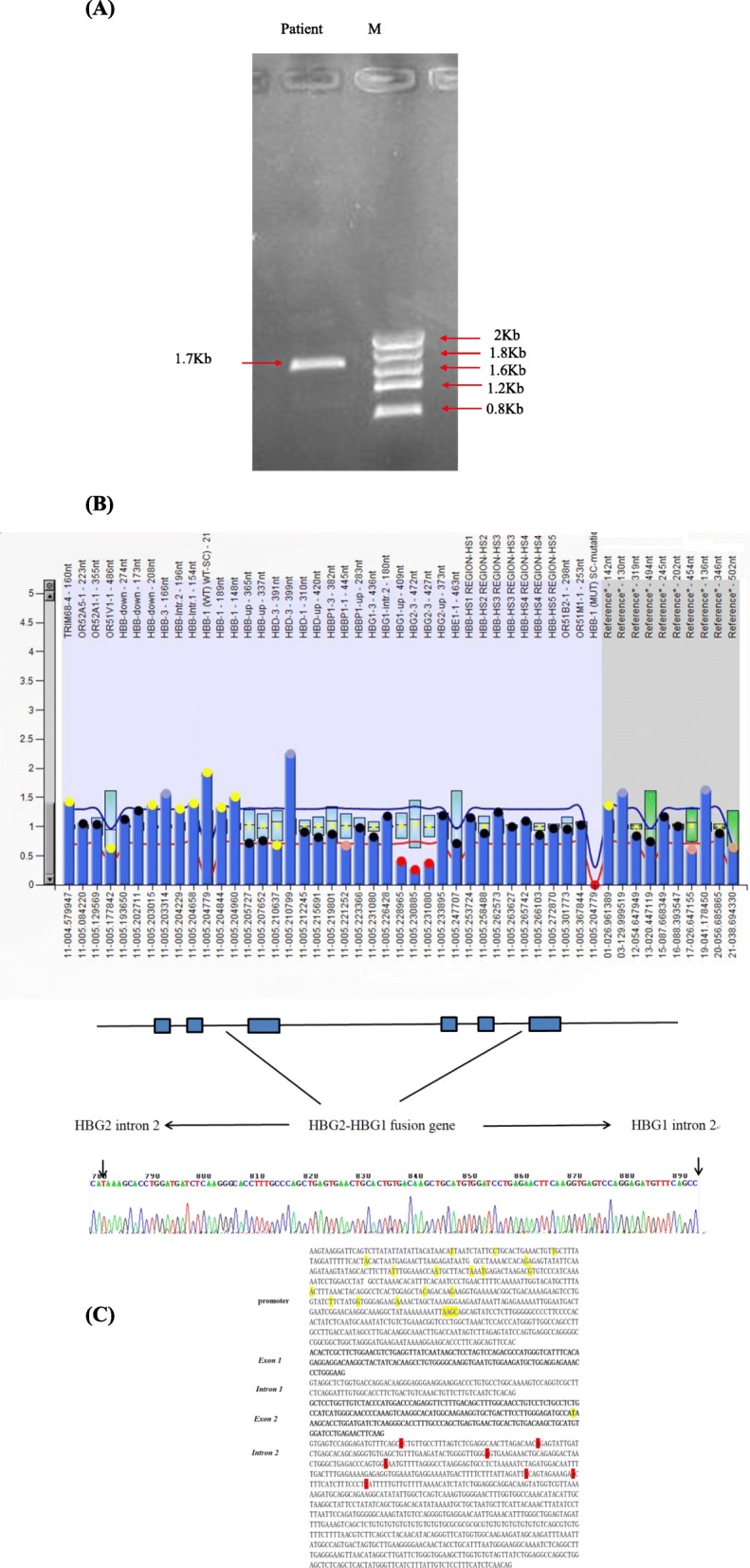
(**a**) PCR primers around probes with normal dosages in the HBG1- IVS2 and HBG2 upstream regions resulting in the amplification of an abnormally shortened PCR product (1.7 kb) in the patient (lane Patient); (**b**) MLPA analysis showing half dosages for three probes located in the HBG2 and HBG1 regions in the patient; (**c**) complete sequences of the HBG2–HBG1 fusion gene in Patient 1. The HBG2 and HBG1 region were composed of two parts: one included the promoter, exon 1, intron 1 and a part of exon 2 of HBG2, and the other included a part of exon 2 and intron 2 of HBG1; HBG2-specific sequences are highlighted in yellow, and HBG1-specific sequences are highlighted in red

**Fig. 7:**
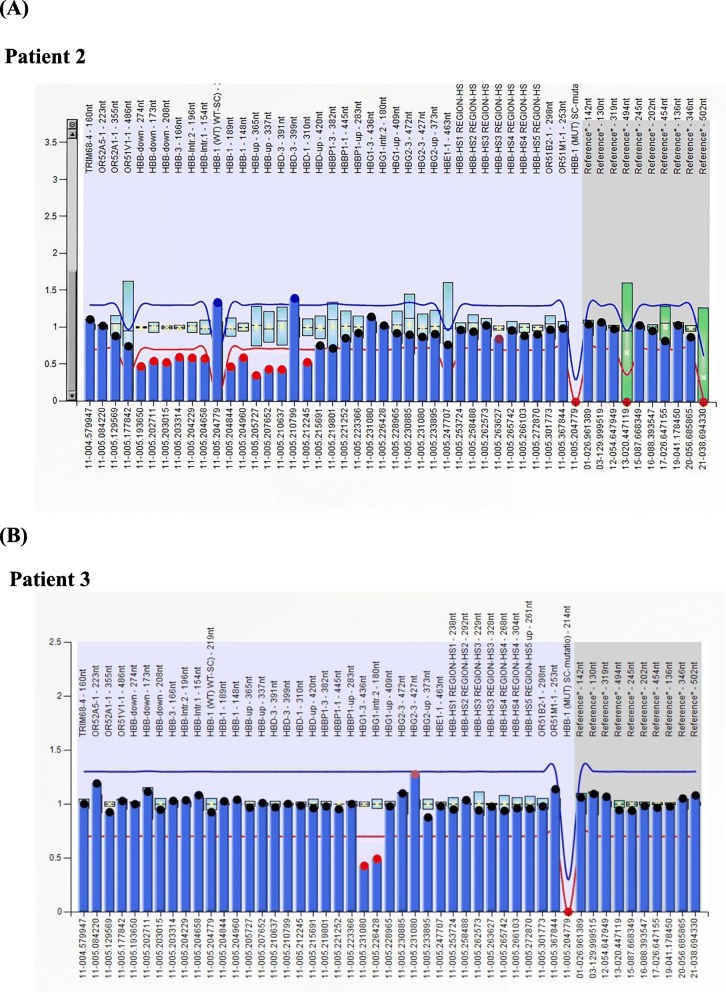
(**a**) MLPA analysis showing half dosages for two probes located in HBG1 in patient 2 and fourteen probes located in the HBB and HBD region in patient 2; (**b**) MLPA analysis showing half dosages for two probes located in HBG1 in patient 3

Three cases of deletions in the β-globin gene cluster were found by MLPA, which has never been reported to date. Case 1, a 30-year-old male, has a low MCV and MCH but a normal hemoglobin level (Hb 162 g/L, MCV 79.1 fL, MCH 28.9 pg). Hb analysis showed increased HbF (9.8%) and lower HbA2 (2.7%) levels. MLPA analysis revealed half dosages for three probes targeting exons 2–3 of HBG2 and the region between HBG2 and HBG1. Primers were designed around probes for which normal dosages were observed, and it yielded a PCR product of 1.7 kb (Fig. 6a). Sequencing indicated a novel deletion from HBG2 exon 2 to the HBG1 intervening sequence (IVS2). A fusion gene from HBG2 and HBG1 was found by specific polymorphisms, but the exact breakpoint could not be determined because of the high homology between HBG2 and HBG (Fig. 6b and c). Case 2, a 31-year-old female with increased HbF (21%) and lower HbA2 (1.8%) levels, had hypochromia and microcytosis (Hb 123 g/L, MCV 76.5 fL, MCH 24.9 pg). MLPA analysis showed half dosages for 13 consecutive MLPA probes with targets ranging from exon 1 of HBD to the 3′-UTR of HBB, indicating δβ-thal (Fig. 7a). Case 3 was a 43-year-old female without other clinical or hematological abnormalities except for a markedly elevated level of HbF (5.8%), the clinical presentation of which was consistent with HPFH.MLPA analysis indicated reduced dosages for two MLPA probes were located in the HBG1 region. All these breakpoints should be further characterized by gap-PCR (Fig. 7b).

## Discussion

Guangzhou is located in the center of southern China and consists of eleven districts. Although the population composition of Guangzhou has changed constantly due to frequent migration from other provinces to this place, people with local residential registration were the largest group in the investigated population, ensuring good representation. This report is the first of its kind to describe the prevalence of (δβ)^0^-thal/HPFH in Guangzhou. Chinese ^G^γ (^A^γδβ)^0^ thalassemia and SEA-HPFH are the most common deletional (δβ)^0^-thal/HPFH disorders in this region. The prevalence of these two disorders was lower than that in the Chinese Zhuang population but higher than that in the Chinese Hakka and Yunnan populations [9–11]. These differences may be caused by the population composition, which is rather homogeneous in those regions. Guangzhou natives primarily include Cantonese and Hakka population, which live scattered in this city, except for the Huadu, Zengchen and Conghua districts. Approximately 30% of the Guangzhou population came from other parts of the province and outside the province. The top five provinces from which immigrants moved were Guangxi, Hunan, Jiangxi, Sichuan, and Hubei. Deletional (δβ)^0^-thal/HPFH has seldom been reported in provinces other than Guangxi, which will have an effect on the distribution of deletional (δβ)^0^-thal/HPFH in Guangzhou. Different distributions of deletional (δβ)^0^-thal/HPFH depend on the composition of the population in 11 districts, especially in the outer suburb and the newly-established district. As a newly-established district, Nansa district provides many preferential policies and facilitation measures to attract people to settle down in this area. The population composition are mainly immigrants.

However, other outer suburbs, such as Huadu district, Guangzhou natives are the main population in these suburbs. In addition, there is a large number of ethnic minorities, including Zhuang and Tujia, in these outer suburbs. Huadu is also the district in which most Hakka people reside. Further research about the effect of ethnic diversity on the carrier rate of these disorders should be conducted by collecting detailed demographic information.

Chinese ^G^γ (^A^γδβ)^0^-thal, SEA-HPFH, the Taiwanese deletion, and the ^A^γ-196C-T mutation are common genetic factors related to high HbF levels in Guangzhou. Only individuals with the ^A^γ-196C-Tmutation had normal values of MCV and MCH. The Taiwanese deletion and Chinese ^G^γ (^A^γδβ)^0^-thal manifested the phenotype of beta^0^-thalassemia, and the values of Hb, MCH and MCV were much lower than normal range. The HbA2 level was lower than 3.5% in heterozygous carriers of Chinese ^G^γ (^A^γδβ)^0^ thal or the ^A^γ-196 C-T mutation, whereas the latter carriers have considerably lower HbA2 levels, regardless of coinheritance with α^0^-thalassaemia (Southeast Asian type deletion(−-^SEA^/αα)) heterozygosity. The mechanism regulating this phenomenon is not clear because the ^A^γ-196C-T mutation only involves a point mutation in HBG1. This mutation is difficult to discriminate among carriers with Southeast Asian type deletion (−-^SEA^/αα) coexistence due to similar hematological indicators. The HbA2 level was higher than 3.5% for cases of heterozygous SEA-HPFH mutation or Taiwanese deletion, and individuals with SEA-HPFH deletion had normal, hypochromic or borderline red blood cell indices. To date, the Taiwanese deletion has only been reported as the case study^,^ [18, 19]. Compared to the three types, that is, Chinese ^G^γ (^A^γδβ)^0^-thal, SEA-HPFH and^A^γ-196 C-T mutation [9, 10, 20], the individual with the Taiwanese deletion showed higher HbA2 levels than 6% of our study cohort. This higher HbA2 level may due to deletion of the β-globin gene promoter. A competitive relationship exists among the β, γ and δ chains in the HS region. ^A^γ-globin gene triplication was observed among our cases, as has been described before. All four of these cases exhibited a high HbF level of more than 8%; in contrast, the HbF level in the case without γ-globin gene triplication was only 3.4%, which was lower than that of the other cases. Whether γ-globin gene triplication contributes to the elevated level of Hb F in the presence of the Taiwan deletion should be further studied. It has been previously described that compound Taiwan deletion with a mutant allele with β-thal generates the β-TI phenotype. Thus, it is necessary to identify Taiwanese deletion carriers in those areas with a high prevalence of thal. The Taiwanese deletion may constitute the best possibility of when no β-globin gene mutation is found in a case of increased HbF and Hb A2 (> 6%).

Only one type of Hb Lepore variant, known as Hb Lepore-Boston/Washington, has been reported in China [21]. Fusion between the HBD gene and the HBB gene occurs due to their high sequence homology. Homozygotes for Hb Lepore or compound heterozygotes for Hb Lepore and β-thalassemia can present with thalassemia major or intermedia. Hb Lepore is highly important for diagnosis, with levels ranging from 5 to 15%.Hb Lepore-Boston/Washington has been described in Thailand [22]. Compared to HPLC (High Performance Liquid Chromatography), the capillary electrophoresis system is a better method for identifying cases with Hb Lepore and Hb E by a peak of denatured Hb E in zone 6, therefore, we can discern compound Hb Lepore/Hb Efrom Hb E/Hb E. It was reported that carriers with Hb Lepore-Boston/Washington showed slightly elevated Hb F levels, which was not consistent with our observations. More cases are needed to illustrate the molecular and hematological characteristics of this disease.

We found a case with the β^− 28^/β Chinese ^G^γ (^A^γδβ)^0^ genotype, a high level of HbF and mild anemia. So CC [23] described a patient with the same genotype who presented moderate anemia. The phenotype of patients with the β^+^/β^Chinese Gγ(Aγδβ)0^ genotype or β^0^/β^Chinese Gγ(Aγδβ)0^ genotype varies, making genetic counseling challenging. There is also some debate regarding pregnancy termination in the case of a fetus with those genotypes.

Three cases of β-globin gene cluster deletions were found by MLPA. Case 1 exhibiting the HBG2-HBG1 fusion gene, was similar to a case reported by Seung-Tae Lee, but the breakpoint may be different [24]. The fusion occurred between HBG2 exon 2 and HBG1 IVS2 in our study, but it consisted of a HBG2-derived 5′ part and a HBG1-derived 3’part in Seung-Tae Lee’s paper. A positive regulatory region,^A^γ-IVS2, has been reported before, and it might influence HbF regulation in adults [25]. The HBG2–HBG1 fusion can cause γ-thalassemia, which has been described before [26]. The breakpoints displayed heterogeneity because the HBG2 and HBG1 genes are highly homologous. The mechanism of γ-thalassemia caused by HBG2–HBG1 gene fusion should be further explored. The functional study of HBG2–HBG1 gene fusion may be performed in K562 cells and HUDEP-2 cells exhibiting embryonic/fetal and adult patterns respectively.

Nondeletional-HPFH has not been described systematically in China. Italian nd-HPFH (^A^γ-196 C-T mutation) was first described in 1984 [20], but it has seldom been reported ever since that. Our study found that Italian nd-HPFH was the most common nondeletional HPFH in Guangzhou, of which the familial prevalence was 0.042%. Because Italian nd-HPFH and Chinese ^G^γ (^A^γδβ)^0^-thal carriers have similar HbF and HbA2 levels, the red blood cell count is the only difference used for discrimination during initial screening for thalassemia. We first detected one Italian nd-HPFH heterozygous condition coexisting α-thal and β-thal, with high HbA2 and HbF levels being detected (HbA2:4.1%, HbF: 25.9%), which is similar to SEA-HPFH carriers. Overall, the phenotypic characteristics of Italian nd-HPFH are useful for genetic counseling for individuals with increased HbF. We also found another nd-HPFH, known as Cretan HPFH (^A^γ-197 C-T), which was described in 2014 [16]. The two types of nd-HPFH are related to point mutations at approximately − 200 relative to the transcriptional start site of the fetal γ-globin gene. Using K562 cells and HUDEP-2 cells, Martyn et al. revealed that mutations at − 200 of the γ-globin promotor disrupt ZBTB7A for HbF repressor binding [27]. These researchers introduced c.–195C > G into HUDEP-2(Δ ^G^γ) WT cells by CRISPR–Cas9 and found that fetal hemoglobin protein levels increased to approximately 24.0%. The results indicated that homozygosity of engineered HPFH cells could cause higher HbF levels than patients with heterozygous HPFH-associated mutations. It was indicated that introducing naturally occurring variants in this region may be considered as an attractive gene therapy strategy. Coincidentally, we detected two homozygotes for the ^A^γ-196 C-T mutation that, to the best of our knowledge, had not been reported previously, raising the possibility that the HbF levels of homozygotes can be similar to those of heterozygotes. Our study is the first to describe a patient in which the binding of ZBTB7A is disrupted on both alleles. There may be some causes for these differences: ZBTB7A may work with additional cofactors at different stages of cell differentiation; the degree to which ZBTB7A influences HbF levels is related to erythropoietic stress, which is characteristic of β-globin chain deficiency. More research is needed to estimate the impact of homozygous and heterozygous ^A^γ-196 C-T mutations on HbF levels, especially when coexisting with major β-thal. If the effect is similar, we can achieve the expected treatment result simply by introducing heterozygous HPFH-associated mutations.

## Conclusions

In summary, our study systematically demonstrated the prevalence and molecular epidemiological characteristics of the hereditary persistence of fetal hemoglobin and δβ-thalassemia in the Greater Guangzhou Area, the capital of Southern China. This is the first report that Italian nd-HPFH (^A^γ-196 C-T mutation) is not rare but in fact the most common nd-HPFH in China. Characterizing phenotypes and genetic mechanisms contribute much to understanding the globin switch and genetic therapy.

## Supplementary information

**Additional file 1.** (A) Hb analysis showing Hb Lepore variant using capillary electrophoresis; (B) MLPA analysis showing half dosages for probes located in the region ranging from exon 3 of HBD to intron 1 of HBB; (C) A representative gel electrophoresis for Hb Lepore-Boston carrier: 915 bp Hb Lepore-Boston specific and 775 bp internal control.

## Data Availability

The datasets generated and/or analysed during the current study areavailable in the [https://pan.baidu.com/] repository, [https://pan.baidu.com/s/1wrsewrPUFjs4OrMaYDNv9A], access code: ajcu. Usename: canliao683; password: jiangfan713.

## Abbreviations

CE: Capillary electrophoresis
Gap-PCR: Gap polymerase chain reaction
Hb A2: Hemoglobin A2
Hb CS: Hb Constant Spring
Hb F: Fetal hemoglobin
Hb QS: Hb Quong Sze
Hb WS: Hb Westmead
Hb: Hemoglobin
HPFH: Hereditary Persistence of Fetal Hemoglobin
HPLC: High Performance Liquid Chromatography
IVS: Intervening sequences
MCH: Mean corpuscular hemoglobin
MCV: Mean corpuscular volume
MLPA: Multiplex ligation-dependent probe amplification
nd-HPFH: Nondeletional HPFH
SEA: Southeast Asian
THAI: Thailand
thal: Thalassemia
β^N^: β^Normal^
β-TI: β-thalassemia intermedia
δβ-thalassemia: Delta beta thalassemia
