# Supplementary material for: Molecular epidemiology and hematologic characterization of δβ-thalassemia and hereditary persistence of fetal hemoglobin in 125,661 families of greater Guangzhou area, the metropolis of southern China
Source: BMC Med Genet. 2020 Feb 28;21:43. doi: 10.1186/s12881-020-0981-x (PMC7049201; doi:10.1186/s12881-020-0981-x)
Supplement: Supplementary file 1 — Additional file 1. (A) Hb analysis showing Hb Lepore variant using capillary electrophoresis; (B) MLPA analysis showing half dosages for probes located in the region ranging from exon 3 of HBD to intron 1 of HBB; (C) A representative gel electrophoresis for Hb Lepore-Boston carrier: 915 bp Hb Lepore-Boston specific and 775 bp internal control. [file 12881_2020_981_MOESM1_ESM.ppt]

## Slide 1
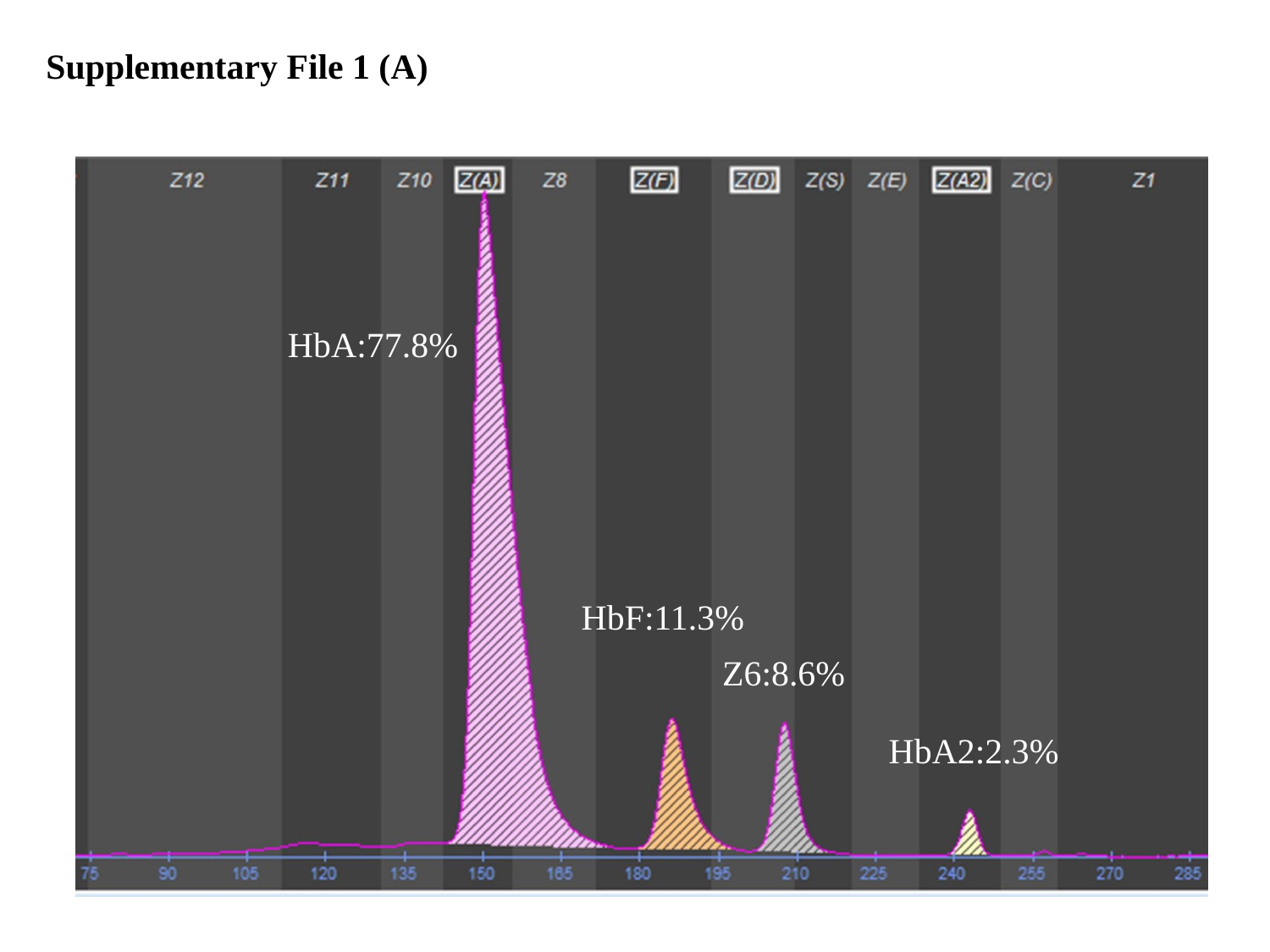

Supplementary File 1 (A)
HbA
HbF(11.6%)
HbA:77.8%
HbF:11.3%
Z6:8.6%
HbA2:2.3%

## Slide 2
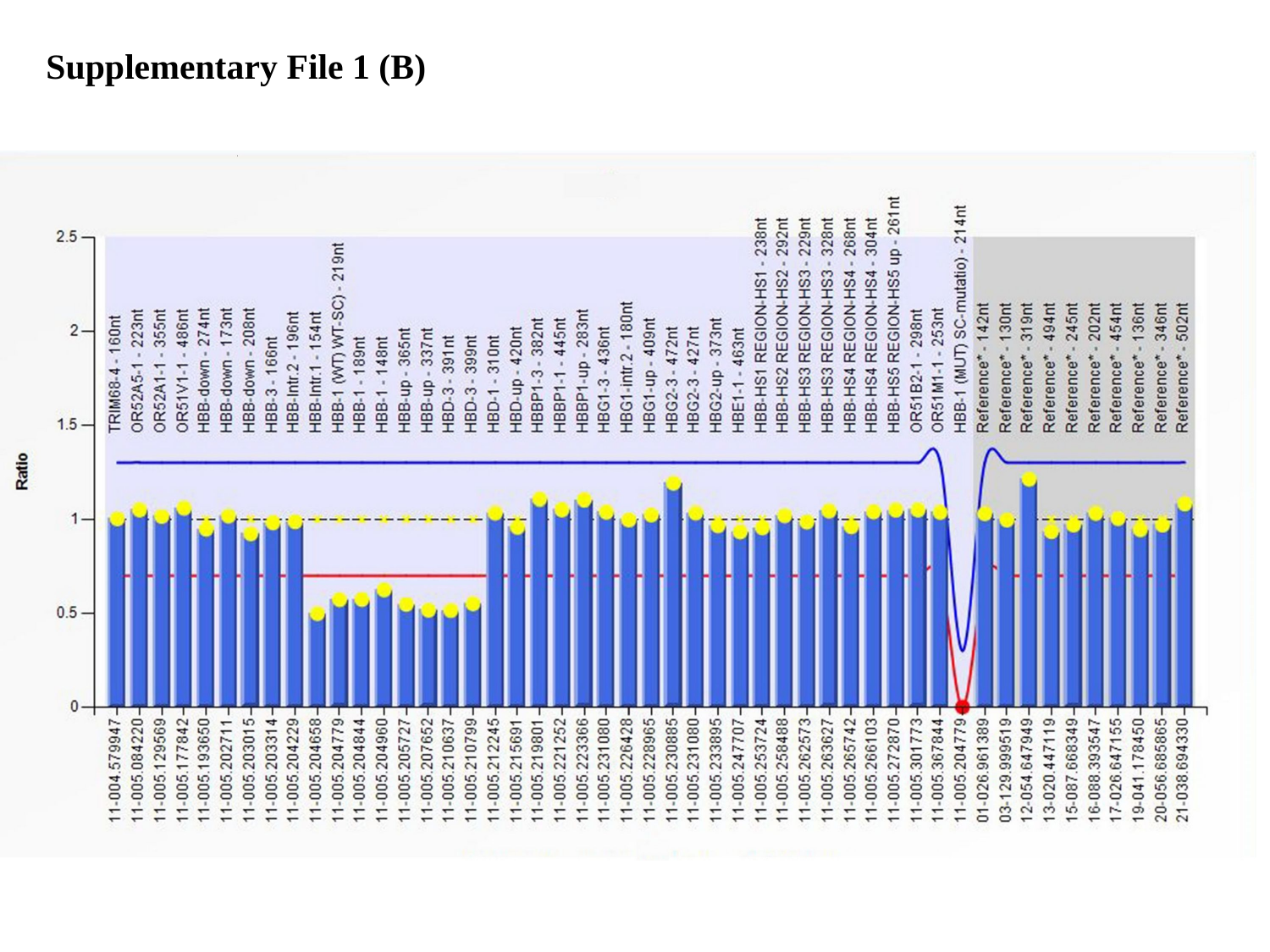

Supplementary File 1 (B)

## Slide 3
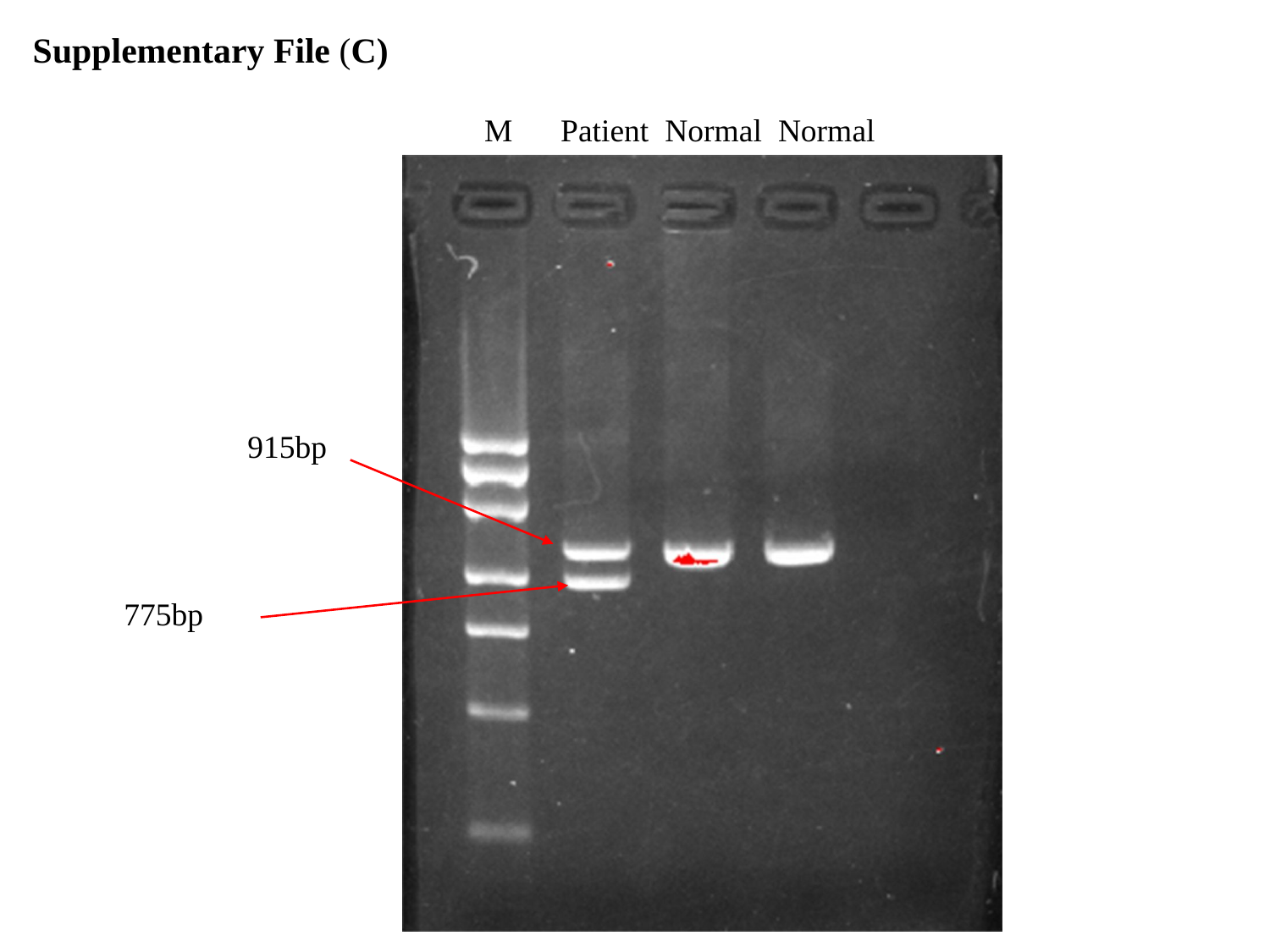

Supplementary File (C)
 M Patient Normal Normal
915bp
775bp
